# EHMTI-0141. URB937 as a potential therapeutic option for migraine: evaluation in animal model of migraine

**DOI:** 10.1186/1129-2377-15-S1-F27

**Published:** 2014-09-18

**Authors:** R Greco, T Bandiera, AS Mangione, F Siani, G Nappi, G Sandrini, A Guijarro, A Armirotti, D Piomelli, C Tassorelli

**Affiliations:** 1 Laboratory of Neurophysiology of Integrative Autonomic Systems Headache Science Centre, "C. Mondino" National Neurological Institute, Pavia, Italy; 2 Drug Discovery and Development, Istituto Italiano di Tecnologia, Genova, Italy; 3 Laboratory of Functional Neurochemistry Center for Research in Neurodegenerative Diseases, "C. Mondino" National Neurological Institute, Pavia, Italy; 4 Dept of Brain and Behaviour, University of Pavia, Pavia, Italy

## Introduction

Systemic nitroglycerin (NTG) activates cerebral nuclei of rat involved in nociceptive transmission, as well as in neuroendocrine and autonomic functions. These changes are considered relevant for migraine pain, since NTG consistently provokes spontaneous-like migraine attacks in migraineurs. Several reports have suggested the existence of relations between the endocannabinoids and migraine. URB937, a peripheral fatty acid amide hydrolase (FAAH) inhibitor, induces analgesia in animal models of pain but there is no information on its effects in migraine.

## Aim

In this study, we evaluated whether the URB937 administration modulates c-Fos expression following NTG administration in specific brain areas of rat.

## Methods

The analgesic effect of URB937 was evaluated in male Sprague Dawley rats. Animals were treated with NTG (10mg/kg, i.p.) followed by URB937 (1mg/kg i.p.) or vehicle (DMSO, 1ml/kg i.p.) and their brain processed for the detection of c-Fos protein. The principles of the Helsinki declaration and IASP's guidelines for pain research in animals were rigorously applied.The experimental research on animals was approved by ethics committee for research on animals of the University of Pavia

## Results

Brain mapping of nuclei activated by NTG administration demonstrated that peripheral FAAH inhibition with URB937, significantly reduces neuronal activation in the nucleus trigeminalis caudalis (NTC) and locus coeruleus (LC).

## Conclusions

These findings show that URB937 may counteract the activation of nuclei involved in migraine attacks probably via the increase of anandamide levels at the meningeal level, within the trigeminovascular system, or on extracerebral vessels.

No conflict of interest.

